# A Comparative Cross-Sectional Study of Coagulation Profiles and Platelet Parameters of *Schistosoma mansoni*-Infected Adults at Haik Primary Hospital, Northeast Ethiopia

**DOI:** 10.1155/2022/5954536

**Published:** 2022-06-27

**Authors:** Habtye Bisetegn, Daniel Getacher Feleke, Hussen Ebrahim, Melkam Tesfaye, Alemu Gedefie, Yonas Erkihun

**Affiliations:** ^1^College of Medicine and Health Sciences, Department of Medical Laboratory Sciences, Wollo University, Dessie, Ethiopia; ^2^Department of Microbiology, Immunology and Parasitology, College of Health Sciences, Addis Ababa University, Addis Ababa, Ethiopia

## Abstract

**Introduction:**

*Schistosoma mansoni* is an intravascular parasite that interacts with all components of the host blood. Nearly, 10% of S. mansoni-infected patients progress to severe hepatosplenic Schistosomiasis is characterized by periportal fibrosis, obstruction of intrahepatic veins, presinusoidal portal hypertension, and splenomegaly. Thus, this study aimed to compare the basic coagulation profiles and platelet parameters of *S. mansoni-*infected adults and noninfected individuals as controls at Haik Primary Hospital, Northeast Ethiopia.

**Methods:**

A comparative cross-sectional study was conducted at Haik Primary Hospital from April to June 2021. The diagnosis and intensity of *S. mansoni* infection was determined using the Kato–Katz technique. The coagulation profiles and platelet parameters were analyzed using coagulation and hematology analyzers. Data were analyzed using SPSS version 26.0. The Kolmogorov–Smirnov and Shapiro–Wilk tests were done to check the distribution of continuous variables. The Mann–Whitney *U* test was used to compare the coagulation profiles and platelet parameters. Spearman's rank-order correlation was done to assess the correlation between the intensity of infection and coagulation profiles and platelet parameters. In all comparison, a *P* value <0.05 was considered statistically significant.

**Result:**

In this study, a total of 180 study participants (90 *S. mansoni*-infected adults and 90 controls) were included. Of the total *S. mansoni*-infected adults, 55.6%, 28.9%, 33, and 15.6% had light, moderate, and heavy intensity of infections, respectively. All *S. mansoni*-infected study participants had prolonged prothrombin time (PT) and international normalized ratio (INR). Moreover, about 80% of *S. mansoni*-infected adults had prolonged activated partial thromboplastin time (APTT). Thrombocytopenia was found in 26.7% of the *S. mansoni*-infected adults. The Mann–Whitney *U* test showed a statistically significant difference in coagulation profiles between *S. mansoni*-infected adults and healthy controls (*P*-value ≤0.001). The Kruskal–Wallis H-test showed a significant difference in PT, APTT, and INR between the intensity of infection and healthy controls (*P*-value <0.05).

**Conclusion:**

This study showed a prolonged coagulation time in *S. mansoni*-infected individuals. Thus, screening of schistosomiasis patients for hemostatic abnormalities and treating the underlying disorder is crucial.

## 1. Introduction

Schistosomiasis is one of the neglected tropical diseases caused by the genus *Schistosoma*. It is the third most devastating tropical disease that accounts for up to 70 million disability-adjusted life years (DALYs) [1]. Globally, an estimated 230 million cases are reported so far with about 800 million people at risk of infection. Majority of these cases occur in sub-Saharan Africa [1–3]. In sub-Saharan Africa, around 393 million people are at risk of infection with 192 million cases [4]. Schistosomiasis kills more than 250,000 people in sub-Saharan Africa alone each year [5, 6].

Several factors contributed to the continuous and persistent transmission of schistosomiasis in sub-Saharan Africa that includes climatic changes, global warming, proximity to water bodies, irrigation, dam construction, occupational activities, and poverty [7]. The high risk group to human schistosomiasis are pre-school children, school-aged children, and people with occupations that involve contact with water that contain the cercarial stage of schistosomes such as irrigation workers, fishermen, farmers, and women [8]. The transmission of schistosomiasis started when infected individuals contaminate freshwater sources with their excreta containing parasite eggs, which later hatch in water and become miracidia [9]. The infective stage for humans is the cercarial stage that penetrates skin during contact with contaminated freshwater [9, 10]. The Kato–Katz stool examination and urine filtration techniques that detect eggs in stool and urine using microscope are the conventional methods used for detection of schistosomiasis [9].

*Schistosoma mansoni* affects the platelet function by the action of kallikrein-like activity (Sk1) [11] which cleaves the plasma protein kininogen to generate the small vasoactive peptide bradykinin which induces the release of prostacyclin (PGI2) from endothelial cells that inhibits platelet degranulation [12]. *S. mansoni* proteases SmCalp1 and/or SmCalp2 that are capable of cleaving the host blood clotting protein fibronectin to impede blood clot formation around the worms in vivo [13]. Obstructions in blood vessels, such as those caused by the presence of the relatively larger adult schisotosomes in the smaller mesenteric venules, and platelet aggregation lead to the disturbances of blood flow and this situation predisposes to thrombotic complications [14]. The live intravascular stages of the parasite produce SmATPDase1 which degrades the prothrombotic nucleotides ATP and ADP; this leads to the inhibition of the coagulation pathway by inhibition of platelet aggregation and thrombus formation [15]. Patients with advanced hepatosplenic schistosomiasis often have abnormalities in hemostasis and fibrinolysis. These include prolongation of prothrombin time (PT) and activated partial thromboplastin time (APTT) as well as thrombocytopenia, hypofibrinogenemia, and decreases of vitamin-K-dependent factors, which relate to the low degree of disseminated intravascular coagulation [16–18].

In Ethiopia, schistosomiasis is a major public health problem with 5.01 million people being infected and 37.5 million people being at risk of infection [19]. *Schistosoma mansoni* is the predominant cause of schistosomiasis in Ethiopia. Several studies were conducted on the prevalence of *S. mansoni* in different endemic areas of Ethiopia [20–24]. Understanding the impact of *S. mansoni* on the human coagulation system and platelet parameters are vital for planning, monitoring, and evaluating strategies to control schistosomiasis-associated morbidity and mortality. So far, there is no study on the impact of *S. mansoni* on the human coagulation system and platelet parameters in the study area. Therefore, this study aimed to provide comprehensive evidence about the impact of *S. mansoni* on coagulation profiles and platelet parameters among *S. mansoni*-infected adult's schistosomiasis at Haik Primary Hospital, Northeast Ethiopia.

## 2. Materials and Methods

### 2.1. Study Area, Design, and Period

This study was conducted at Haik Primary Hospital located in Haik town, Northeast Ethiopia.

Haik town is found at 430 Km from the North of Addis Ababa, the capital of Ethiopia. The town is located at 130 30.59″N latitude and 039028.849″E longitude. Its altitude is 2200 meter above sea level and the area covers 447.8 square kilometer with a total population of 108,993. The major occupations of the inhabitants include trade, civil service, daily labor, and subsistence agriculture in the suburban villages. There are water bodies in and around Haik town that includes Ankerca River, Logo Lake, Ardibo Lake, and Kette River. There was a study that reported the endemicity of *S. mansoni* infection in the study area [23].

This institution-based comparative cross-sectional study was conducted at Haik Primary Hospital, Northeastern Ethiopia, from April to June 2021.

### 2.2. Eligibility Criteria

#### 2.2.1. Inclusion Criteria

Individuals examined microscopically for *S. mansoni* infection using the Kato–Katz technique and found infected with *S. mansoni* attending Haik Primary Hospital during the study period and who are willing to give written consent for their participation were included in this study. *Schistosoma mansoni* negative blood donors at the Dessie blood bank who volunteer to participate in the study were included as a control group.

#### 2.2.2. Exclusion Criteria

Individuals who are chronic alcohol drinkers, regular chat chewer, current cigarette smokers, viral hepatitis B and C seropositive, HIV/AIDS patients, pregnant women, anticoagulant or antiaggregant drugs users, and those suffering from chronic diseases apart from schistosomiasis were carefully excluded.

Patients were also excluded if they are presented with splenectomy, diabetes mellitus, hypertension, use of hepatotoxic drugs, thrombocytopenic drugs, or drugs that the change platelet function (such as acetylsalicylic acid), chronic renal diseases, and lactating mothers. Patients with a history of malignancy and inherited bleeding disorder were also excluded.

### 2.3. Sample Size Determination and Sampling Technique

According to the rule of thumb recommended by van Voorhis and Morgan, 30 participants per group are required to detect real differences that could lead to about 80% power [25]. In the present study, more study participants were recruited to increase the reliability of the study. Thus, a total of 180 (90 *S. mansoni*-infected adults and 90 apparently healthy controls) were enrolled. The simple random sampling technique was used to recruit study participants.

### 2.4. Data Collection and Laboratory Investigation

Sociodemographic characteristics of the study participants were collected using pretested questionnaires by the investigators and trained data collectors.

Clinical information and physical diagnosis of *S. mansoni*-infected adults and apparently healthy comparators were assessed by physicians and trained nurses. Laboratory investigations of study participants for some of the infectious diseases were also done to check eligibility.

### 2.5. Sample Collection and Processing

A stool sample was collected from each *S. mansoni* suspected patient using leak-proof containers.

*S. mansoni* diagnosis was done by direct wet mount microscopic examination of the stool using normal saline (0.85% sodium chloride solution). The intensity of infection was determined using the Kato–Katz technique. Blood sample was collected from each study participant and healthy controls following standard operating procedures (SOPs) by trained laboratory technologists. About 5.7 ml of the blood sample was collected with a sterile disposable syringe. Then, about 2.7 ml of the collected blood sample was delivered into a citrated tube and mixed properly without any frothing. After mixing gently, the platelet-poor plasma was obtained by centrifuging at 1500 g for 15 minutes for APTT and PT analysis. The remaining 3 ml of blood was dispensed into an EDTA tube for the determination of platelet parameters.

### 2.6. Platelet Parameters and Coagulation Analysis

The methods of Hussen Ebrahim et al. were used to analyze the coagulation and platelet parameters [26]. Platelet parameters (platelet count, mean platelet volume (MPV), and platelet distribution width (PDW)) were analyzed using a Dirui BF 6500 automated hematology analyzer (Dirui, China) within 2 hours of sample collection. Furthermore, APTT, PT, and international normalized ratio (INR) were determined using a coagulation analyzer (Huma Clot Junior, Germany). The first 50 microliters (*μ*l) test plasma was warmed at 37°C for 5 minutes. At the same time, the APTT reagent and CaCl2 were simultaneously incubated. Then, 50 *μ*l APTT reagent was added to the warmed platelet-poor plasma and incubated at 37°C for 3 minutes, followed by the addition of 50 *μ*l prewarmed CaCl2 buffer solution. The coagulation analyzer read the clotting time of APTT and displayed the result in seconds. One hundred *μ*l of the test platelet-poor plasma was added into the test cuvette and incubated for 3 minutes at 37°C. Subsequently, 200 *μ*l of the prewarmed PT reagent was added and the time taken for clot formation (in seconds) was recorded, and at the same time, INR was calculated and displayed from the PT result.

### 2.7. Statistical Analysis

Data were entered to Epi Data version 3.1 and exported to SPSS version 26.0 for analysis.

The Kolmogorov–Smirnov and Shapiro–Wilk normality tests were done to assess distribution of continuous variables. The Mann–Whitney *U* test was used to compare the coagulation profiles and platelet parameters of *S. mansoni*-infected adults and noninfected individuals. Spearman's rank-order correlation was done to assess the correlation between the intensity of infection and coagulation profiles and platelet parameters. In all comparison, a *P* value <0.05 was considered statistically significant.

### 2.8. Quality Assurance

The quality of the blood sample was maintained by collecting and processing it according to the standard operating procedures (SOPs). Samples were checked for the absence of hemolysis and clotting, sample volume, collection time, and correct labeling. Safety and specimen handling procedures were strictly followed. The performance of the coagulometer was checked by the daily running of two-level controls (normal and high). The performance of the hematology analyzer was monitored by daily background checking. The quality of the Kato–Katz examination was checked daily and 10% of the slides were randomly selected and re-examined at the end by an experienced laboratory technologist who was blind for the first examination result.

## 3. Result

### 3.1. Sociodemographic Characteristics

A total of 180 study participants consisting of 90 *S. mansoni*-infected adults (48 males and 42 females with an age range between 18 and 62 years) and 90 noninfected adults (49 males and 41 females with an age range between 19 and 54 years) were involved. The mean ± standard deviation (SD) age of the S*. mansoni*-infected adults and healthy controls were 30.33 ± 12.26 and 31.2 ± 12.85 years, respectively. Generally, the *S. mansoni*-infected adults and healthy controls are not significantly different in terms of sociodemographic characteristics. Among *S. mansoni*-infected participants, about 60% of them were urban residents (Table 1).

### 3.2. Basic Coagulation Profiles and Platelet Parameters of the Study Participants

All the *S. mansoni*-infected study participants had prolonged PT and INR. Similarly, about 80% of the *S. mansoni*-infected participants had prolonged APTT. On the other hand, thrombocytopenia was found in 26.7% of *S. mansoni*-infected adults. Moreover, the MPV was lower in 83.3% of the *S. mansoni*-infected adults. Majority of the coagulation profiles and platelet parameters were found normal in *S. mansoni* noninfected individuals (Table 2).

### 3.3. Comparison of the Coagulation Profile and Platelet Parameter among *S. mansoni*-Infected Adults and Noninfected Controls

The median and interquartile range (IQR) of PT and APTT of *S. mansoni*-infected participants were 17.1 (2) and 37.8 (3) seconds, respectively. The Mann–Whitney *U* test showed a statistically significant increase in PT, INR, APTT, and PDW among *S. mansoni*-infected adults compared to apparently healthy controls (*P*-value ≤0.001). The median platelet count among *S. mansoni*-infected adults was lower than that among noninfected individuals. However, the difference was not statistically significant (Table 3).

### 3.4. Intensity of Infection and Its Correlation with Coagulation Profiles and Platelet Parameters

The overall median of egg per gram (EPG) of stool in the *S. mansoni*-infected patients was 215 EPG. Of the total *S. mansoni*-infected adults, 55.6% (95%CI: 44.4–65.6), 28.9% (95%CI: 20– 23138.9), and 15.6% (95%CI: 8.9–23.3) had light, moderate, and heavy intensity of infections, respectively. Spearman's rank-order correlation showed a statistically significant and positive correlation between the intensity of *S. mansoni* infection and PT, APTT, INR, and platelet count with a correlation coefficient of 0.43, 0.255, 0.210, and 0.231, respectively, with a *P* value <0.05. The Kruskal–Wallis H-test showed also showed a statistically significant difference in PT, APTT, and INR values between the intensity of *S. mansoni*-infected individuals and *S. mansoni* noninfected individuals (Table 4).

## 4. Discussion

Schistosomiasis is a neglected tropical disease that affects more than 240 million people in tropical and subtropical countries. It is responsible for more than 70 million disability-adjusted life years [3].

The present study aimed to investigate the impact of *S. mansoni* infection on the coagulation system and platelet parameters at Haik Primary Hospital, Northeast Ethiopia. The median and interquartile range (IQR) of platelet count of *S. mansoni*-infected participants was 244(152) × 103/ul. This finding was similar with a study conducted in Saudi Arabia [27]. 

In the present study, thrombocytopenia was observed in 26.7% of *S. mansoni*-infected participants. The low platelet count in *S. mansoni*-infected adults might be due to the massive adhesion of platelets to the *S. mansoni* eggshell in the vascular endothelium [28]. It has been also reported that *S. mansoni*-infected patients produce anti-schistosome antibodies that crossreact with platelet and this leads to clearance of the platelet and which in turn causes thrombocytopenia [29].

This study also revealed that all the *S. mansoni*-infected patients had prolonged PT and INR with a median and interquartile range (IQR) of 17.1(2) and 1.79 (0.19) seconds, respectively. Moreover, about 80% of the *S. mansoni*-infected participants showed prolonged APTT with a median and interquartile range (IQR) of 37.8 (3) seconds. This finding was in agreement with a report from Brazil that found APTT of 37.96 ± 1.5 seconds [16]. Impairment of the coagulation system or the prolonged PT, INR, and APTT in the *S. mansoni*-infected individuals could be due to various mechanisms carried out by the parasite. There are a number of mechanisms that cause impairment of the coagulation system. These includes depletion of platelet count, presence of the large-sized adult worm in the blood vessel that leads to obstruction of the vessel and disturbance of blood flow, degradation of the prothrombic nucleotide by the live parasites, impediment thrombin-driven platelet activation, reduced synthesis of clotting factors, inhibition of the proteolytic activity of thrombin, inhibition acting on factor XIIa, consumption of plasma clotting protein, and presence of heparin-like glycosaminoglycans on the worm tegument that interferes with hemostasis. Moreover, *S. mansoni* APT diphosphohydrolase 1(smAPTDase1) degrades host prothrombotic signals like APT and ADP and block platelet activation and blood coagulation [13–15]. The median (IQR) MPV and PDW were 6.05 (1.6) and 17.6(3.5) fl, respectively. The MPV was lower than the normal value in 83.7% of S*. mansoni*-infected adults. Moreover, PDW was high in 43.3% and low in 10% of the *S. mansoni*-infected individuals.

The increase in PT, INR, and APTT among S*. mansoni*-infected adults was statistically significant when compared to *S. mansoni* noninfected individuals (*P*-value ≤0.001). Moreover, in the present study, the platelet parameters (MPV and PDW) were also significantly different between *S. mansoni*-infected adults and healthy controls (*P* value ≤ 0.001). This finding was similar to studies done in Northwest Ethiopia [30], in Brazil [16], and Western Burkina Faso [31]. The MPV was significantly decreased among *S. mansoni*-infected adults compared to healthy controls. Furthermore, this study revealed that RDW was significantly increased among *S. mansoni*-infected adults compared to healthy controls.

Spearman's rank-order correlation showed that there was a significant and positive correlation between the intensity of *S. mansoni* infection and PT, APTT, INR, and platelet count with a correlation coefficient of 0.43, 0.255, 0.210, and 0.231, respectively, with a *P* value <0.05. The Kruskal–Wallis H-test showed that there was a significant difference in PT, APTT, and INR values between the intensity of infection and apparently healthy controls. This finding was in line with the report from Sanja Primary Hospital, Northwest Ethiopia [30]. The difference in platelet parameters between healthy controls and the intensity of *S. mansoni* infection in adults was not statistically significant.

## 5. Conclusion

The present study showed that the coagulation time among *S. mansoni*-infected patients is prolonged. This indicates that screening of schistosomiasis patients for hemostatic abnormalities and treating the underlying disorder has paramount importance. This finding also alerts the clinicians to screen schistosomiasis patients for underlying hematological tests before proceeding to any surgical procedures. Finally, the authors would like to recommend besides the treatment of *S. mansoni*-infected patients with praziquantel, the patients have to be screened and treated for schistosomiasis-associated hematological, hemostatic, and biochemical abnormalities. The prevention and control of *S. mansoni* infection should also be strengthened and implemented well.

## Figures and Tables

**Table 1:** Sociodemographic characteristics of *S. mansoni*-infected and noninfected study participants.

Variables		*S. mansoni positive* (*N* = 90)	Control (*N* = 90)
Mean age and standard deviation		30.33 ± 12.26	31.2 ± 12.85

Sex	Male	48 (53.3%)	49 (54.4%)
	Female	42 (46.7%)	41(45.6%)

Residence	Urban	54 (60%)	50 (55.6)
	Rural	36 (40%)	40 (44.4%)

Educational status	Illiterate	27 (30%)	28(31.1%)
	Primary	24 (26.7%)	22(24.4%)
	Secondary	24 (26.7%)	23(25.6%)
	College and above	15 (19.7%)	17(16.7%)

Occupation	Student	27 (30%)	27(30%)
	Government employee	9(10%)	11(12.2%)
	Farmer	12 (13.3%)	13(14.4%)
	House wife	27(30%)	25(27.8%)
	Private	15(19.7%)	16(17.8)

**Table 2:** Basic coagulation profiles and platelet parameters of the study participants and healthy controls.

Variables		*S. mansoni*-infected adults (*N* = 90)	Healthy controls (*N* = 90)	Reference range
		Frequency %	Frequency %	
PT	Shortened	0	1 (1.1)	14–16 sec
	Normal	0	87 (96.7)	
	Prolonged	90 (100)	2 (2.2)	

INR	Shortened	0	0	0.8–1.2
	Normal	0	86 (95.6)	
	Prolonged	90 (100)	4 (4.4)	

APTT	Shortened	0	12 (13.3)	24–36 sec
	Normal	18 (20)	78 (86.7)	
	Prolonged	72 (80)	0	

Platelet count	Low	24 (26.7)	0	150–400^*∗*^103/*μ*l
	Normal	60 (66.7)	90 (100)	
	High	6 (6.7)	0	

PDW	Low	9 (10)	10 (11.1)	15–18 fl
	Normal	42 (46.7)	78 (86.7)	
	High	39 (43.3)	2 (2.2)	

MPV	Low	75 (83.3)	0	7–13 fl
	Normal	15 (16.7)	90 (100)	
	High	0	0	

PT: prothrombin time, APTT: activated partial thromboplastin time, INR: international normalization ratio, PDW: platelet distribution width, MPV: mean platelet volume, *μ*l: microliter, fl: femtoliter.

**Table 3:** Comparison of coagulation profiles and platelet parameters among *S. mansoni*-infected adults and noninfected individuals controls

Variables	*S. mansoni*-infected adult *N* = 90	Healthy controls *N* = 90	*P*-value
	Median (IQR)	Median (IQR)	
PT (seconds)	17.1 (2)	13.9 (1.7)	**≤0.001**
INR	1.79 (0.19)	1.15 (0.14)	**≤0.001**
APTT (seconds)	37.8 (3)	23.4 (5.1)	**≤0.001**
Platelet count (10^3^/*μ*l)	244 (152)	277.0 (75)	0.215
PDW (fl)	17.6 (3.5)	15.6 (1.0)	**≤0.001**
MPV (fl)	6.05 (1.6)	9.7 (1.2)	**≤0.001**

*N* = 180, PT: prothrombin time, APTT: activated partial thromboplastin time, INR: international normalization ratio, PDW, platelet distribution width, MPV: mean platelet volume, *μ*l: microliter, fl: femtoliter.

**Table 4:** Comparison of coagulation profiles and platelet parameters between the intensity of *S. mansoni*-infected individuals and noninfected individuals (Kruskal–Wallis H-test).

Variables	Healthy controls	*S. mansoni*-infected adults			*P* value
		Light infection (1–99 EPG)	Moderate infection (100–399 EPG)	Heavy infection (≥400EPG)	
PT (seconds)	13.9 (1.7)	16.3 (1.6)	17.2 (1.45)	18.4(1.43)	**≤0.001**
INR	1.15 (0.14)	1.69 (0.24)	1.8 (0.17)	1.85(0.1)	**0.014**
APTT (seconds)	23.4 (5.1)	36.7 (2.95)	38.9 (2.13)	39.4(2.3)	**0.045**
Platelet count (103/*μ*l)	277.0 (75)	225.5 (130)	222.4 (128)	220(124)	0.282
PDW (fl)	15.6 (1.0)	16.58 (2.58)	18.15 (4)	17.4(2.35)	0.055
MPV (fl)	9.7 (1.2)	6 (1.45)	5.9 (2.46)	4.5(2.31)	0.976

## Data Availability

All the data required for this research are available within the manuscript. If additional data are needed, it can be obtained from the corresponding authors upon request.

## Abbreviations

APTT: Activated partial thromboplastin time
INR: International normalized ration
MPV: Mean platelet volume
PDW: Platelet distribution width
SOP: Standard operating procedure
PT: Prothrombin time.
